# Microsaccadic modulation evoked by emotional events

**DOI:** 10.1186/s40101-020-00238-6

**Published:** 2020-09-04

**Authors:** Koji Kashihara

**Affiliations:** 1 College of Information Science and Engineering, Ritsumeikan University, 1-1-1 Noji-higashi, Kusatsu, Shiga 525-8577 Japan; 2 Graduate School of Technology, Industrial and Social Sciences, Tokushima University, 2-1 Minamijyousanjima, Tokushima, 770-8506 Japan

**Keywords:** Emotional attention, Small eye movements, Microsaccadic direction, Brain mechanism, Mental illness

## Abstract

Saccadic eye movements can allude to emotional states and visual attention. Recent studies have shown that microsaccadic responses (i.e., small fixational eye movements) reflect advanced brain activity during attentional and cognitive tasks. Moreover, the microsaccadic activity related to emotional attention provides new insights into this field. For example, emotional pictures attenuate the microsaccadic rate, and microsaccadic responses to covert attention occur in the direction opposite to a negative emotional target. However, the effects of various emotional events on microsaccadic activity remain debatable. This review introduces visual attention and eye movement studies that support findings on the modulation of microsaccadic responses to emotional events, comparing them with typical microsaccadic responses. This review also discusses the brain neuronal mechanisms governing microsaccadic responses to the attentional shifts triggered by emotion-related stimuli. It is hard to reveal the direct brain pathway of the microsaccadic modulation, especially in advanced (e.g., sustained anger, envy, distrust, guilt, frustration, delight, attraction, trust, and love), but also in basic human emotions (i.e., anger, disgust, fear, happiness, sadness, and surprise). However, non-human primates and human studies can uncover the possible brain pathways of emotional attention and microsaccades, thus providing future research directions. In particular, the facilitated (or reduced) attention is common evidence that microsaccadic activities change under a variety of social modalities (e.g., cognition, music, mental illness, and working memory) that elicit emotions and feelings.

## Background

### Fundamental functions of microsaccades

Staring at a target causes small eye movements such as microsaccades, drifts, and tremors. Microsaccades are the fastest and largest of the small eye movements and travel in a straight trajectory. Drifts are slow curvy motions that occur between microsaccades. Tremors are highly fast and small oscillations superimposed on drifts [1]. Microsaccadic activity during fixation on a stationary target plays an important role in preventing loss of vision by shifting the retinal image [1]. Microsaccadic responses are also correlated with advanced brain activities such as visual and/or auditory perception and cognition [2, 3]. The microsaccadic rate is improved by attention, visual cues that imply succeeding target appearance [4], and oddball tasks [5, 6]. Moreover, hearing meaningful words [7], absorption in listening to music [8], and mental fatigue [9], which are related to eliciting specific emotions and feelings, can modulate microsaccadic responses. Spatial attention to an emotional target (e.g., a negative or positive image) induces voluntary saccades with increased fixation time [10, 11]. The attention to centrally or peripherally presented emotional pictures changes the microsaccadic rate and direction [12]. However, whether the various social modalities that induce emotional changes in our daily life can cause microsaccadic modulation remains uncertain.

### Visuospatial attention and microsaccades

Before describing the cases of emotional events, visuospatial attention can be generally understood as follows. Attention to a spatial location is classified into overt and covert attention (i.e., with and without eye movement toward a target), and attention orientation involves endogenous and exogenous attention [13, 14]. Endogenous attention (e.g., visual or auditory cues associated with attentional shifts) arises from a voluntary top-down process originating in internal states and expectations. By contrast, exogenous attention (e.g., a sudden burst of sound or blight light) is due to an involuntary bottom-up process controlled by external sensory events [15]. Individuals under covert attention can monitor the surrounding environment—an activity suggesting subsequent eye movements triggered by sustained endogenous or temporary exogenous attention [16]. Such covert attention can cause a dynamic microsaccadic response: an acute drop and subsequent enhancement [4, 17]. Furthermore, visual or auditory oddball tasks reduce the appearance of microsaccades during the rebound process [5, 6, 18]. Emotion-related attention can also depict similar microsaccadic activity. On the other hand, attentional tasks using simple cues such as arrows, color, and flash appear to minimally hinder such appearance [4, 19].

### Biased microsaccadic directions

Microsaccadic directions are biased toward or away from the spatial location indicated by an attentional cue [4, 18, 20, 21]. Central cues that attract endogenous attention can evoke microsaccadic biases toward an implied spatial location. The microsaccadic direction corresponds to the direction of the attentional shift triggered by spatial cues, and such direction shows an indicator of covert attention [4, 20]. Contrastingly, abrupt and salient stimuli for engaging exogenous attention enhance microsaccadic biases in the direction opposite to a target place [19, 22]. This can be described by the mechanism of “inhibition of return” [23] in covert orienting [17, 24]. However, whether microsaccadic direction can imply emotional attention (i.e., the attention induced by emotional stimuli such as pictures and sounds) associated with our social modalities remains controversial.

### Objectives

Microsaccades during basic activities and their physiological mechanisms have been excellently summarized in previous reviews (e.g., [1, 25, 26]). This review has therefore focused on the microsaccadic activity in response to emotion-related attention and its cognitive and brain mechanisms, especially in attention orienting and inhibitory control.

## Microsaccadic responses to emotional attention

### Prolonged microsaccadic inhibition

#### Emotional pictures

**Fig. 1:**
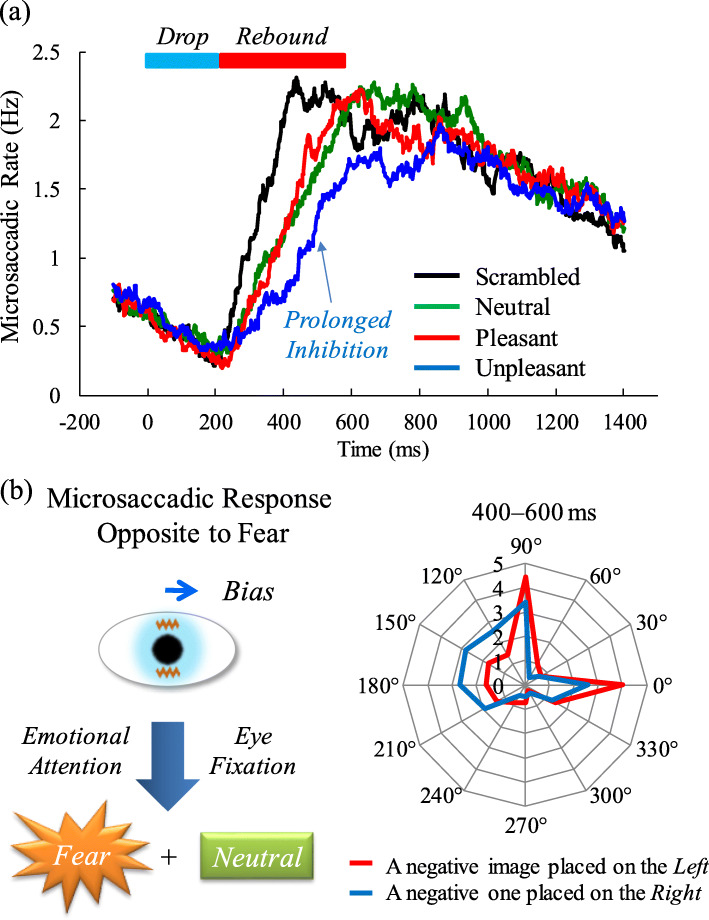
Fundamental understanding of microsaccadic activity in response to emotions. **a** Changes in microsaccadic rates under the presentation of emotional pictures: an initial drop period followed by a rebound period. The inhibited rebound process of the negative emotional condition was statistically significant compared to that of the scrambled, neutral, or positive condition [12], although the microsaccadic rate during the rebound was more than that of the prestimulus period. **b** Direction of the microsaccades (the average number of appearance) during the rebound period (400–600 ms) in response to negative pictures to the left (*red* line) or right (*blue* line) side [12]

The International Affective Picture System (IAPS) [27] dataset can induce covert (or overt) emotional attention and change microsaccadic activity (i.e., appearance rate and direction) [12]. The microsaccadic responses to emotional (neutral, positive, and negative) pictures resulted in rapid inhibition (0–200 ms) and succeeding rebound (200–600 ms) after stimulus presentation: the biphasic (inhibition-rebound) response (Fig. 1a). A persistent possibility is that the initial lack of microsaccades after emotion-related stimuli can act as the common preparation process for the succeeding rebound process, which is similar to those after visual or auditory stimuli [22, 28]. In particular, microsaccadic appearance during the rebound period was attenuated by attention to negative rather than positive emotional pictures [12]; for the average rating scores of the valence, arousal, and dominance in the IAPS used in this study, the change from the neutral category was greater in the negative than positive category. Usually, the arousal level in the IAPS similarly becomes higher in the unpleasant valence (especially for fear) [29]. Thus, there are some difficult cases in which the selected IAPS dataset cannot universally induce an extremely high positive emotion among participants. The recent study [30] by Krejtz et al. supports that the aversive stimuli based on the IAPS can be easy to modulate the microsaccadic response.

From the results mentioned above, it is speculated that the degree of microsaccadic inhibition during the rebound period may depend on the attention level to an emotional event which is characterized by individual arousal, valence, and dominance. Many people similarly experience negative emotions during situations that appear life-threatening (e.g., feeling threatened by an animal, witnessing violence, and car accidents). However, a rare group of people may not be as affected by negative emotional events (e.g., fans of horror films), and some cases show a low arousal level even when looking at negative pictures (e.g., depression and sadness [31]). Most people can commonly feel positive when looking at pretty animals and babies. By contrast, owing to a variety of individual preferences (e.g., favorite sports, food, and hobbies) [12, 29], it may be difficult to select positive emotional events that induce great attraction universally. Thus, it will be easier for an image dataset to substantially change microsaccadic responses upon attention toward negative emotions. However, further investigations are required to assess everyday emotional events, with consideration of the individual differences (e.g., preferences, gender, and aging) [32–34] in how positive and negative scenes and actions are received.

**Fig. 2:**
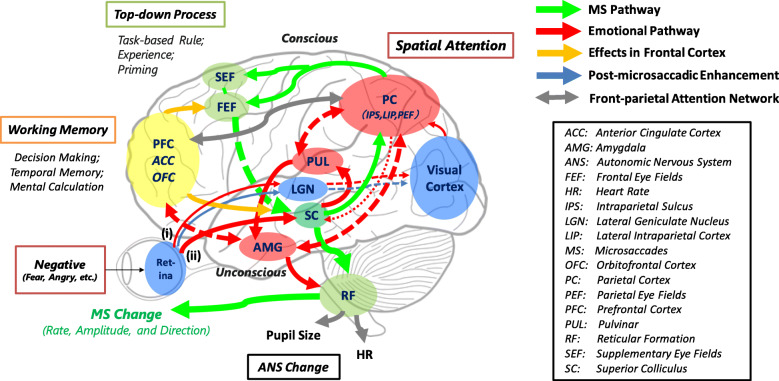
Possible brain neuronal mechanisms of the microsaccadic activity modulated by the spatial and negative emotional attention. Microsaccadic production (*green* lines) (e.g., [25]) and emotional attention (*red* lines) (e.g., [35–37]) pathways. Emotional inputs can be divided into two pathways: (i) cortical and (ii) subcortical routes. *Dotted* lines indicate cortico-subcortical pathways. Note that the possible interactive pathways for emotional attention and microsaccades were limited within references for this review, although there exist various kinds of pathways

For cognitive or attentional tasks, cortical centers (Fig. 2) can control microsaccadic inhibition during a rebound period [5, 6]. The microsaccadic rebound process also plays an important role in preventing visual fading and restoring perception [1, 38]. It is, therefore, speculated that suppressed rebound responses to emotional stimuli [12] can retain emotional information at the expense of an updating process for visual input, using early brain processing through a subcortical pathway activated by emotional attention [39]. During this rebound process, attentional resources are presumably allocated to detect emotional events, rather than generating small eye movements for resetting afterimages [1]. Contrast levels and non-informative cues (e.g., colors and symbols) can also produce biphasic microsaccadic responses [4, 19]. However, the microsaccadic rate during the rebound is more strongly diminished by negative emotional pictures than by scrambled and neutral ones, even with the same luminance [12], implying that the various types of social or emotion-related attention can also modulate microsaccadic activity.

#### Clinical diseases

Clinical diseases and symptoms become useful indicators to explicate the normal functioning of healthy people. Since mental illnesses, especially mood and anxiety disorders, suggest damaged or weakened emotional controls, the changes in microsaccadic activity under psychiatric disorders (e.g., [40]) can indicate the necessary functions for emotional and oculomotor controls in healthy people. More recently, Yep et al. [41] reported that facial (i.e., neutral, happy, sad, fearful, and angry) expressions caused the microsaccadic suppression (100–150 ms after the onset of face stimuli) and succeeding rebound (250–300 ms) in the normal subjects and the groups with attention-deficit hyperactivity disorder (ADHD) and bipolar disorders. The time-course (inhibition-rebound) changes of this study were very similar to those in other attention/cognitive tasks for microsaccades (e.g., [4, 5]). However, the microsaccadic rebound period was shorter than those in the IAPS (200–600 ms [12]), meaning taking a long time to treat with affective IAPS stimuli.

The exogenous stimuli under visual oddball paradigms can instantaneously elicit reflexive attention with a strange, novel, or surprising feeling, presumably needing a long period to inhibit the rebound microsaccadic response (e.g., around 200–700 ms [5]; 200–400 ms [6]). This response closely resembles that of the IAPS data [12], rather than basic facial expressions [41], and this is supported by the evidence that the average rating score of the arousal is lower among facial expressions than the typical IAPS dataset [29]. The quick rebound process in facial expressions is close to that in a burst sound (100–250 ms [28]), which may be transient and slight emotional changes compared to the IAPS.

According to the report by Yep et al. [41], the peak of the microsaccadic rebound tended to decrease in mentally ill individuals (i.e., bipolar disorder rather than ADHD or control subjects), indirectly implying the significant effects of emotional elements on microsaccadic responses, even in healthy people. Furthermore, the microsaccadic rate for fearful stimuli preceding prosaccade trials was attenuated among the group with bipolar disorders, rather than the ADHD group. A point to notice is that such mental illnesses have specific features. For example, patients with bipolar disorders indicate prefrontal-subcortical network dysfunction and are slower to identify fearful faces [42]. Individuals with psychiatric disorders perceive neutral faces as ambiguous or negative [43]. These features in patients may give a hint to elucidate the mechanism of microsaccadic responses.

#### Facial expressions

More importantly, Yep et al. [41] assessed the executive functions using the pro/anti saccadic responses to a peripheral target with a centrally presented emotional face. The ages of the participants were varied, consisting of both young and elderly people, who were required to respond to a correct target “removing” an emotional face, in accordance with a saccadic rule based on a presented facial gender. This condition may divide the attentional resources into executive function and emotional processing. As described above, the previous microsaccadic studies have mainly focused on priming cues followed by a target selection task to induce covert attention, or oddball tasks to induce exogenous attention (e.g., a strange or different sound at a rare rate). Compared to those studies, the executive function test with facial expressions will require higher operations: cognitive and emotional processing. Therefore, those additional or complex factors may cause no or slight effects on the inhibited microsaccadic rebound by facial expressions.

#### Various social modalities

Lange et al. [8] found that absorption by listening to music changed the microsaccadic rate, suggesting that more emotional music (i.e., valence and arousal changes) could influence microsaccades. Furthermore, hearing meaningful words induced microsaccadic inhibition during a rebound period (around 400 to 800 ms after the stimulus onset), compared to pseudowords [7]. These results suggest that the microsaccadic modulation is strongly associated with social modalities such as listening to music and hearing words that induce deeper emotions. Winterson and Collewun [44] observed that microsaccadic inhibition occurred during high-acuity tasks, such as threading a needle and sighting-in a rifle.

By contrast, Ko et al. [45] reported that the microsaccadic rate was increased during a high-acuity needle-threading task, which required high accuracy and long-term attention/concentration (around 15 s) to perform spatial adjustment, compared to the typical dynamics of fixational eye movements (< 1 s). Thus, the absorption or attention/concentration is a common key factor in revealing the various microsaccadic dynamics. Because emotional actions can also shift covert attention, the change in microsaccadic activity will depend on the valence and arousal factors to determine the degree of emotional attention. Interestingly, the microsaccadic rates decreased with the increase of attentional demand [46] and task difficulty in mental arithmetic [47], which can elicit difficult feelings. However, Benedetto et al. [48] stated that the microsaccadic rate increased during a driving task with a secondary searching task (i.e., the dual task), compared to the baseline and control. In addition to the factors highlighted by previous studies (e.g., [8, 9, 41]), attention which is greatly facilitated by sustained, vigorous, and deep emotional reactions may be able to modulate microsaccadic responses.

### Microsaccadic direction

#### Inhibition-of-return effects

When endogenous covert attention is induced by a central cue that implies the next target’s direction, the microsaccadic appearance is biased toward the same direction [4, 20]. In contrast, peripheral cues are biased away from a target in longer latency [19, 24]. The reports by Galfano et al. [17] and Betta et al. [24] give support to the notion that the suppression of a reflexive saccade by a peripheral event induces an opposite microsaccadic bias. Using the experimental results and the computational modeling in a cueing task, Tian et al. [23] have proposed that microsaccades are constantly biased opposite to the cue, in order to stabilize retinal image, after the initial cue-directed response, as sustained, but not transient, effects.

#### Emotional input

The microsaccadic responses during a rebound period were biased toward the direction opposite to an emotional image that induced exogenous covert attention [12] (Fig. 1b). This bias can be regarded as a kind of inhibition-of-return effects in microsaccades, indicating a performance drop at the spatial location of exogenous attentional salience. Because subliminally negative stimuli of peripheral cues show the inhibition-of-return effects [49], it is speculated that these effects in emotions modulate the microsaccadic direction. In addition to lower sensory activity to elicit exogenous attention, such as illuminance and colors, more complicated neuronal networks may be included for emotional perception or recognition, suggesting interactive effects on microsaccadic production by emotional brain pathways (Fig. 2).

When neutral and emotional scenes are simultaneously shown at peripheral sides, the initial fixation that succeeds saccadic eye movements is projected on an emotional picture [50, 51]. Therefore, the microsaccadic responses to the opposite direction [12] may result from the retention of central fixation, which is aimed at preventing the activation of voluntary saccades against the direction of emotional image appearance. Such directional biases may also counterbalance the generation of microsaccades [24], inhibiting their rate of appearance during the rebound period [12]. Godlove and Schall [52] found that significantly fewer microsaccades were generated when monkeys successfully canceled normal saccades, and the few microsaccades escaping this inhibition showed a tendency to be directed toward the target place. These results suggest that the prolonged microsaccadic inhibition is due to the operation of saccade cancelation (Fig. 1), which may be related to the attentional capture or the inhibition of return [53]. Moreover, the lower dominance of negative pictures may help to release the attention from a target and generate anti-microsaccades.

#### Absence of microsaccades

Microsaccadic biases were unremarkable during the early period (100–200 ms) after the onset of the emotional picture [12]. Turatto et al. [21] reported that the bias opposite to the location of a salient event occurs during a discrimination period but not during a detection period. Thus, stimulus detection at a low sensory level (e.g., the luminance of pictures), rather than the semantic interpretation of a presented emotional image, may cause the early absence of microsaccadic bias. Although facial expressions affected microsaccadic rates, there were no significant differences among facial expressions in a pro/anti-saccade task [41]. The directional biases of microsaccades remain unknown under facial stimuli. However, the IAPS dataset (e.g., fear and anger) could induce the microsaccadic biases more easily because those pictures show the greater changes in arousal as well as valence levels [29].

## Brain mechanisms for emotional attention and microsaccades

### Prolonged microsaccadic inhibition

#### Major brain pathways

The possible brain pathways for microsaccadic modulation due to emotional inputs are shown in Fig. 2: microsaccade generation (e.g., [25]) and emotional attention (e.g., [35–37]) pathways. Emotional stimuli (*red* lines in Fig. 2) can change microsaccadic activity (*green* lines) through the brain’s neuronal networks, primarily in the superior colliculus (SC), the frontal eye fields (FEF), the pulvinar (PUL) projecting to the amygdala (AMG) and the parietal cortex (PC), and the prefrontal cortex (PFC). These pathways are firmly associated with emotion [16, 39, 54], as well as the SC and FEF modulation during the fixational eye movement [1, 55–57]. The SC can drive microsaccadic activities, such as the initial inhibition of microsaccades related to the disruption of stable visual perception [55, 56, 58]. Immediately after this initial inhibition, the rebound process that underlies the microsaccadic rate is acutely facilitated, resulting in the removal of an afterimage and the prevention of the fading of useless information. If the visually perceived information is crucial (e.g., impending fear or dangerous scenes), microsaccadic activity may be suppressed even during a rebound period, to preserve the obtained information.

#### Control of microsaccades

Peel et al. [57] assessed the role of the FEF in the cortical oculomotor area of monkeys, which is projecting to the SC in the generation of microsaccades, resulting in the critical top-down control, especially during the recovery process of microsaccades. The FEF area also cooperates with the PC (lateral intraparietal area mainly in primates) for spatial attention [25, 26]. Therefore, the cortical activities of the FEF and PC regions crucial for the top-down process can reflect the SC (Fig. 2) [59], presumably linking to the inhibited or enhanced microsaccadic rate. On the other hand, although the prior microsaccadic generation can increase the neural activity in the lateral geniculate nucleus, the primary visual area, and the extrastriate cortex, the early vision system is almost irrelevant to the inhibitive actions [26, 38, 60]. The retinal or extraretinal signals can bring about such post-microsaccadic enhancement of neuronal firing rates in the early vision system [25, 26, 61]. These brain areas appear to contribute to preserving vision and preventing visual fading, rather than reflecting advanced brain functions, even during emotional attention.

#### Control of emotion

The brain neuronal activity to emotional inputs can be basically classified into two major pathways (Fig. 2) [35]. First, the endogenous (voluntary) attention to emotional stimuli activates the cortical areas such as the FEF and the PC [36]. The emotion-related top-down attention in such cortical regions might change the activity of the subcortical regions crucial for microsaccadic generation owing to the tight relationship between both the areas. Secondly, emotional inputs rapidly stimulate the AMG through subcortical pathways such as the SC and the PUL [39, 62]. As a defensive behavior, the AMG can trigger a freezing response to potentially dangerous events [63]. Fear-related reflexes also pass the neuronal route between the AMG and the SC [54]. These results imply that a sudden negative event can activate the subcortical route, and the microsaccadic appearance originating in the SC may be interrupted because of fear-induced freezing or the attentional resources distributed for directional biases.

In bipolar patients with an abnormal activation of the right AMG [64] in response to fearful faces, the dysregulated limbic system circuitry involving the anterior cingulate cortex and the orbitofrontal cortex (OFC) may also affect the microsaccadic modulation [41]. Furthermore, social-cognitive and emotional processing requires the shared brain pathway and complicated interactions [65, 66]. When emotional cognitive and attentional tasks require the saccadic preparation and execution, the interactive brain activity can be activated between the oculomotor and subcortical emotional circuits (e.g., PFC, FEF, supplementary eye fields, parietal eye fields, and thalamus) [67, 68].

#### Limited speculation

There is limited speculation regarding the microsaccadic modulation by emotional stimuli. Even if there is not a direct pathway to change the general microsaccadic response, the emotional inputs and processing could reduce the attentional resource for microsaccadic activity, presumably inhibiting the rebound response. The total attentional resource to complete emotional cognitive tasks should be distributed separately between the microsaccade generation and emotional attention loops. This can reduce the usual activity of microsaccades. For instance, some attentional resources must be spent removing the irrelevant obstacles (e.g., exogenous emotional stimuli) for a target task, the remaining resources, which are supposed to work with spatial attention, may be attenuated during the microsaccadic rebound or enhanced process.

The PUL is the primary brain area to remove obstacles and shift attention to the next target [69]. At the same time, this area is a key terminal area to control emotions as a subcortical pathway [35]. It is, therefore, speculated that the separately distributed attentional resources to carry out multiple operations simultaneously can cause the absence of microsaccadic responses because of strongly activated pathways in emotions, such as the AMG and the PUL. As an example of positive emotions, the pedunculopontine tegmental nucleus activity in monkeys, with attentive and high arousal conditions during a reward-biased task, may facilitate the production of fixational saccades, as a gate for saccade signals to the SC [70]. Future works should address the brain functional differences between animal primates and humans, especially in the cortical areas related to advanced emotions.

### Microsaccadic direction

The opposite-side response to emotional attention during a rebound period is biased toward the left eye field in right-side image appearance [12]. For voluntary saccadic activity, a signal pathway from the FEF to the SC can inhibit eye movements that indicate a vector toward a target location [71]. Accordingly, keeping eye fixation may counteract voluntary saccadic activity toward visual salience and induce microsaccadic bias in the opposite direction. During the top-down process, microsaccadic activity also involves the parietal lobe and the FEF [26, 72]. Given that a right hemispheric bias is observed during emotional perception and expression and that the right-hemisphere reflects bilateral visual fields [73, 74], the function of the right visual field can be accelerated, even in right-side emotional target appearance [75]. This acceleration increases the anti-microsaccadic responses necessary to retain eye fixation. Definitively identifying the brain mechanism responsible for the acceleration necessitates further investigation into the interactions between microsaccades and emotion.

The effects of attention to an auditory target on the direction of microsaccades have been confirmed only among left-sided cues (i.e., unilateral direction [22]). Since this result is inconsistent with that of emotional attention [12], it can be argued that the type of attentional stimuli (i.e., visual or auditory) may alter the microsaccadic direction. Horizontally directed microsaccades can be based on the brainstem activity responsible for saccade generation [5, 19, 76]. By contrast, the microsaccadic bias toward the vertical (upward or downward) direction acts on compensation for opposite-side drift [77]. The microsaccadic direction may be also involved in the act of accessing working memory in an emotional place.

As described above, in endogenous covert attention, microsaccades tend to be directed toward the target, as the capturing attention related to the saccade planning for a subsequent reaction. In exogenous attention, they are directed away from the target, as the inhibition of return [25]. The SC contributes to the saccade for the capturing attention or the inhibition of return [53, 78] and even plays a crucial role in the microsaccadic regulation, as well as the FEF [79]. Therefore, the inhibition-of-return effects observed even in microsaccades [17, 23, 24] also have a high possibility to use the SC and FEF regions. The SC is connected to the reticular formation [25], where a final command is directly output to the oculomotor nerve to induce saccades (or microsaccades).

However, this SC area has a complex interaction because of the terminal concentrated from various cortical sites (i.e., the top-down process) and the basic attentional circuit and sensory areas in the limbic system (i.e., the bottom-up process) especially during the cognitive and emotional processing, resulting in bringing about confusion in this field. It is also speculated that the PUL-cortical interaction for attentional release [69] might affect the directional biases in microsaccadic responses to emotional events sharing such areas [35]. Although the PUL nucleus is a key structure for saccades and visual attention functions [80], its function is unclear in microsaccades.

## Microsaccades and emotional attention in physiological anthropology

### Visual perception

Visual perception of detecting motion provides a crucial benefit for the evolution of visual systems, especially in predator*–*prey interaction. For example, a frog can catch the changes in a rapidly moving fly, whereas the frog loses sight of the resting fly [81]. Fixational eye movements make it possible to activate visibility and prevent the fading of stationary targets. Such eye movements can be observed in a variety of animals such as mammals, birds, fish, reptiles, and amphibians [82].

In the human visual system, the rapid retinal adaptation to detect moving targets might have evolved as a means to survive a severe environment. By contrast, the retinal adaptation for viewing stationary targets induces perceptual fading [1]. For example, a stationary target on which eyes are fixated will gradually disappear into the surrounding images, which is called “Troxler effect” [1]. This response is completed through the neural adaptation of the cones, rods, and ganglion cells in the retina, bringing about an attenuated neuronal reaction to a light stimulus and reduced signal strength to the brain. The human oculomotor system can generate small eye movements to prevent such retinal adaptation to a stationary target [4, 83], implying a trace of evolutionary history.

### Emotional attention

It is important to understand the neuronal processes that take place during attentional orientation in animals and humans. Visual attention plays a key role in controlling saccades [84], and there are some cases in which attentional orientation is different from orientating gaze position (i.e., “covert attention” [13]). The small eye movements (i.e., microsaccades) in human vision reflect covert attention as one of the advanced brain activities as described above. The microsaccades have various visual processes to allocate cognitive and/or emotional attention, driving neurons in the central nervous system to prevent image fading, which may be likened to a “window on the mind” [81].

Rather than refreshing visual perception, the microsaccadic rate is acutely decreased while viewing emotional scenes [12, 30]. This implies that the blunted microsaccadic activity can instantaneously maintain the crucial information needed to protect the individual from danger by temporarily stopping the updating of the vision. At the same time, the emotional attention can elicit pupillary mydriasis as an action of the autonomic nervous system (ANS) [12, 85] to capture the significant emotional scene as much as possible. Such specific characteristics of the oculomotor activity might have been acquired in the process of human evolution. However, the role of microsaccades remains an unsolved evolutionary puzzle.

### Brain mechanisms

The neural network system for visual attention orienting may have been produced by evolutionary history in animals and humans [86, 87]. The midbrain network in vertebrates can reflexively orientate the eyes toward salient events. These reflexive eye movements play a defensive role in ensuring survival, and they can be integrated with cortical neural networks for strategic control. With the evolution of complicated behavior based on experience, motivation, and emotion, the human brain has acquired the flexibility to correspond to a variety of environmental events requiring multiple types of selective attention. This selective attention can be controlled by integrating subcortical reflex circuits and cortical processes [88]. In particular, the microsaccades of the small eye movements can imply the covert attention associated with cognition and emotion thorough highly advanced brain controls (see Fig. 2 for details).

### Perspective

#### Pathophysiology

ADHD [89], schizophrenia [90], and autism [91], elicit dysfunctional oculomotor control. In particular, individuals with bipolar disorders showed a high sensitivity to microsaccadic modulation [41] and illustrated abnormal activation in the PFC, OFC, and AMG [92, 93] strongly related to emotional attention. Thus, the study of miniature eye movements among individuals with mental diseases will facilitate the understanding of the physiological responses and brain connectivity [40] necessary for the normal functioning of microsaccadic activity. Assessing activity during abnormal states such as bipolar disorders [41], post-traumatic stress disorder triggered by a terrifying event, and mental stress or fatigue [9] will provide new insights into the brain mechanism of microsaccadic responses to emotional events by focusing on the damaged brain areas or pathways.

#### Autonomic nervous system

Future directions that are worth exploring include revealing the interactions between microsaccadic responses and other physiological responses. In particular, the ANS activity, such as the changes in heartbeats [94] and pupil size [95], is involved in a part of the complex process of fixational eye movements. Emotional stimuli induce ANS activity such as heart rate change [96] and pupil size variation [85, 97], suggesting an interactional relationship with microsaccadic activity. The changes in pupil size and microsaccadic activity are triggered together when emotional pictures are viewed, and the levels of miosis are more strongly suppressed by emotional than neutral pictures with the same luminance [12].

Furthermore, unexpected flashing during fixation can trigger a reflexive pupillary response that signals the onset of microsaccadic overproduction [95]. The study by Privitera et al. [98] indicated that the ANS activity of pupillary reflex can interact with microsaccadic activity. Owing to the coupling of heartbeats and microsaccadic activities [94], the emotionally changed heart rate may also cause the microsaccadic modulation. On the other hand, there is a report showing that the pupil dilation reveals decision formation, but with few microsaccadic changes [99]. Since the ANS is generally referred to as the involuntary motor system [100], such synchronization as peripheral nerve activities (i.e., pupil dilation and heart rate mainly regulated by the brainstem and thalamus) seems to be basically separated from the microsaccadic pathways mainly including higher central actions such as the FEF, PC, and PFC activities (Fig. 2) [98].

#### Task difficulty

The task difficulty in mental arithmetic can increase the attention level as a priority, inducing decreased microsaccadic rates with increased magnitudes [47]. The different feelings (easy or difficult) during this task, which was confirmed by the rating scores of the Self-Assessment-Manikin (valence and arousal) to assess emotional states and the NASA-TLX as an indicator of perceived task difficulty (i.e., greatly changed scores in the difficult than easy task), induced the microsaccadic modulation. However, an opposite case also exists. Although the mental fatigue and sleepiness induced by a time-on-task (i.e., a simplified air traffic control task) changed microsaccadic dynamics, it was irrelevant to task complexity [9]. In addition to focusing on the difficulty of complex tasks, hybrid tasks such as the affective Go/No-Go task, the emotional Stroop paradigm, and the affective priming task may bring about a further understanding of the interactive activities between microsaccades and emotional attention.

## Conclusions

Emotional attention can inhibit the frequency of microsaccadic appearance, especially during a rebound period. Interestingly, the microsaccadic appearance for covert attention is oriented toward the direction opposite to the negative emotional stimuli during an inhibited rebound period. The neuronal network activity triggered by emotional events could change microsaccadic activity. Given these findings, an important requirement is to investigate microsaccadic activity under various social or emotional situations, including mental disorders. Not only the basic emotions but also various social modalities and bimodal actions linked with complex emotions (e.g., envy, guilt, frustration, attraction, trust, and love) have a possibility to affect the microsaccadic activity, depending on whether the attention process is top-down or bottom-up. From the viewpoints of physiological anthropology, it is important to clarify the potential benefits in human functions, focusing on the unknown mechanisms of small eye movements.

## Data Availability

Not applicable.

## Abbreviations

ADHD: Attention-deficit hyperactivity disorder
AMG: Amygdala
ANS: Autonomic nervous system
FEF: Frontal eye fields
IAPS: International Affective Picture System
OFC: Orbitofrontal cortex
PC: Parietal cortex
PFC: Prefrontal cortex
PUL: Pulvinar
SC: Superior colliculus
